# Comparison of the formula accuracy for calculating multifocal intraocular lens power: a single center retrospective study in Korean patients

**DOI:** 10.1038/s41598-024-54889-x

**Published:** 2024-02-23

**Authors:** Jinchul Kim, Joonsung Park, Yoonjung Jo

**Affiliations:** Department of Ophthalmology, Miracle Eye Clinic, Teheran-ro, Gangnam-gu, Seoul, 06134 South Korea

**Keywords:** Biophysics, Computational biology and bioinformatics, Diseases, Optics and photonics

## Abstract

This study evaluated the accuracy of newer formulas (Barrett Universal II, EVO 2.0, Kane, Hoffer QST, and PEARL-DGS) and the Haigis formula in Korean patients with the Alcon TFNT multifocal intraocular lens. In total, 3100 randomly selected eyes of 3100 patients were retrospectively reviewed. After constant optimization, the standard deviation (SD) of the prediction error was assessed for the entire group, and the root mean square error was compared for short and long axial length (AL) subgroup analysis. The Cooke-modified AL (CMAL) was experimentally applied to the Haigis formula. All the newer formulas performed well, but they did not significantly outperform the Haigis formula. In addition, all the newer formulas exhibited significant myopic outcomes (− 0.23 to − 0.29 diopters) in long eyes. Application of the CMAL to the Haigis formula with single constant optimization produced similar behavior and higher correlation with the newer formulas. The CMAL-applied triple-optimized Haigis formula yielded a substantially smaller SD, even superior to the Barrett and Hoffer QST formulas. The AL modification algorithms such as the CMAL used in newer formulas to cope with optical biometry’s overestimation of the AL in long eyes seemed to overcompensate, particularly in the long eyes of the East Asian population.

## Introduction

Currently, the main quality standard of success in cataract surgery is the postoperative refractive outcome^1,2^, especially when a multifocal intraocular lens (IOL) is implanted^3–5^. Due to higher expectations, deviation of the final refraction from the target is far less forgiving in multifocal IOL implantation than in monofocal IOL implantation^3^.

Moreover, multifocal IOLs using simultaneous images inevitably accompany photic phenomena, which seem to wane with time^6^. Therefore, along with meticulous patient selection^3,7^, leaving minimal residual refractive error would diminish patients’ discomfort, enabling them to neuro-adapt the simultaneous images and fully enjoy a spectacle-free lifestyle^8,9^.

Although patients constantly seek spectacle independence and multifocal IOLs have been in the market for several decades^10^, their popularity seems to have surged recently^11^. The advent of newer multifocal IOLs^10^ and the commendable improvement in IOL calculation accuracy^1^ may have encouraged surgeons to implant multifocal IOLs more confidently.

Since the introduction of the laser interferometer^12^ and its recent evolution to swept-source optical coherence tomography-based biometry^13^, the accuracy of biometric measurements has become less of an issue. The primary source of error lies in effective lens position (ELP) prediction^14^, which, unlike other precisely measurable variables, can only be predicted. Although all theoretical IOL calculation formulas share the same optical backbone^15^, their ELP prediction algorithms differ^16^.

The more stringent standard for postoperative refractive status in multifocal IOL implantation^3^ makes surgeons prone to seek more reliable references. Recently, many novel formulas have emerged and have been increasingly replacing their predecessors^17^. These formulas deliver superior outcomes compared with the classic formulas^2,18–20^. However, more influential large-population studies have primarily reported monofocal IOL results^2,18,20,21^. For multifocal IOLs, reliable references are rare because of the enrolment of a small population^3^, or noncompliance to guidelines, such as including both eyes without appropriate statistical analysis^22–24^.

This study aimed to compare the accuracy of freely accessible novel formulas for a single^23^ multifocal IOL model in a large dataset from a Korean population.

## Results

A total of 3100 eyes from 3100 patients were included.

Table 1 summarizes the patients’ demographic characteristics. Overall, the newer formulas and Haigis formula showed a substantially lower standard deviation (SD) than the Hoffer Q, Holladay 1, and SRK/T formulas (Table 2). As this result was well anticipated^2,15,18,19,21^, these older two-variable formulas, legacies of the pre-optical biometry era, were excluded from further analysis to avoid less-engaging comparisons.

**Table 1:** Demographics of the patient population (N = 3100).

Characteristics	Value	Range
Right eye, n (%)	1586 (51.2)	
Female sex, n (%)	2287 (73.8)	
Short eyes (AL < 22 mm), n (%)	134 (4.32)	
Long eyes (AL > 26 mm), n (%)	133 (4.29)	
Age (years)	58.7 ± 5.7	38–87
IOL power (D)	20.27 ± 3.40	6–31
Axial length (mm)	23.68 ± 1.15	20.57–29.73
Mean conventional keratometry (D)	44.21 ± 1.40	39.63–50.50
Mean total keratometry (D)	44.18 ± 1.40	39.41–50.28
Anterior chamber depth (mm)	3.14 ± 0.35	1.89–4.30
Lens thickness (mm)	4.47 ± 0.31	3.37–5.66
Central corneal thickness (µm)	541.35 ± 32.23	434–685
Corneal diameter (mm)	11.81 ± 0.38	10.5–13.7
Postoperative SE (D)	− 0.086 ± 0.33	− 1.25–1.0
Postoperative BCDVA, LogMAR	0.01 ± 0.03	0.00–0.15
Postoperative UCDVA, LogMAR	0.02 ± 0.05	0.00–0.22
Postoperative UCIVA, LogMAR	0.03 ± 0.06	0.00–0.30
Postoperative UCNVA, LogMAR	0.02 ± 0.06	0.00–0.30

Values are shown as numbers (percentage) or mean ± standard deviation.

*AL* axial length, *BCDVA* best corrected distance visual acuity, *D* diopter, *IOL* intraocular lens, *LogMAR* Logarithm of the minimum angle of resolution, *SE* spherical equivalent, *UCDVA* Uncorrected distance visual acuity, *UCIVA* Uncorrected intermediate visual acuity, *UCNVA* Uncorrected near visual acuity.

**Table 2:** Prediction errors of each formula in the whole group.

Whole group (N = 3100)				
Formula	ME	SD	MedAE	MAE
PEARL	− 2.0E−06	0.3175	0.2040	0.2481
HTCL	− 0.0015	0.3133	0.2011	0.2457
Kane	0.0001	0.3168	0.2031	0.2480
EVO 2.0	− 1.2E−05	0.3171	0.2042	0.2483
HTAL	− 0.0019	0.3221	0.2133	0.2532
HSAL	0.0006	0.3226	0.2115	0.2542
HSCL	2.4E−05	0.3260	0.2109	0.2561
Barrett	0.0002	0.3282	0.2125	0.2577
Hoffer QST	− 0.0001	0.3358	0.2230	0.2660
Holladay 1	− 3.4E−06	0.3505	0.2360	0.2782
Hoffer Q	− 5.8E−06	0.3534	0.2375	0.2805
SRK/T	− 0.0004	0.3902	0.2556	0.3091

The optimized constants for the formulas are: Hoffer Q pACD: 0.571, Holladay 1 SF: 1.859, SRK/T A constant: 119.076. For the Haigis formula, HSAL a0: 1.523, a1: 0.4, a2: 0.1, HTAL a0: 1.304, a1: 0.442, a2: 0.104, HSCL a0: 1.556, a1: 0.4, a2: 0.1, HTCL a0: 3.526, a1: 0.523, a2: 0, PEARL, A constant: 119.27; Kane, A constant: 119.16; EVO 2.0, A constant: 119.15; Barrett, A constant: 119.18; and Hoffer QST, pACD: 5.664.

*AL* axial length, *CMAL* Cooke-modified axial length, *HSAL* Haigis formula, single-optimized and AL-applied, *HSCL* Haigis formula, single-optimized and CMAL-applied, *HTAL* Haigis formula, triple-optimized and AL-applied, *HTCL* Haigis formula, triple-optimized and CMAL-applied, *MAE* mean absolute error, *ME* mean numerical prediction error, *MedAE* median absolute error, *RMSE* root mean square numerical error, *SD* standard deviation, *EVO* Emmetropia Verifying Optical formula, *Hoffer QST* Hoffer Q/Savini/Taroni formula, *PEARL–DGS* Prediction Enhanced by Artificial Intelligence and output Linearization–Debellemanière Gatinel, and Saad.

The Kane, EVO 2.0, and PEARL-DGS formulas exhibited similar levels of accuracy. The Haigis formula with both single and triple optimizations ranked next. The discrepancy between the three best-performing newer formulas and the conventional AL-applied Haigis formula (both single- and triple-optimized) was not statistically significant (P > 0.05, Table 3). The Barrett formula did not outperform the Haigis formula and significantly underperformed compared to the more recently developed formulas, except for the Hoffer QST formula, which utilizes a smaller number of variables^25^ (Tables 2, 3). All novel formulas performed excellently in the short eye subgroup (see Supplementary Table S1).

**Table 3:** Statistical comparison of the standard deviation of the formulas for the whole group with adjusted *P*-values (heteroscedastic test and Holm correction).

Formula	HTCL	Kane	EVO 2.0	PEARL	HTAL	HSAL	HSCL	Barrett
HTCL	–	–	–	–	–	–	–	–
Kane	0.8411	–	–	–	–	–	–	–
EVO 2.0	0.8411	0.8411	–	–	–	–	–	–
PEARL	0.8411	0.8411	0.8411	–	–	–	–	–
HTAL	0.E+00*	0.5824	0.6286	0.7645	–	–	–	–
HSAL	0.E+00*	0.0739	0.5824	0.7488	0.8411	–	–	–
HSCL	**5.3E**–10*	0.0003*	0.0034*	0.0013*	0.3744	0.5716	–	–
Barrett	0.0003*	8.2E−06*	2.9E−14*	2.9E−05*	0.4920	0.5824	0.8411	–
Hoffer QST	2.8E−13*	5.7E−14*	0.E+00*	3.0E−09*	2.7E−06*	4.0E−06*	0.0014*	0.0091*

*AL* axial length, *CMAL* Cooke-modified axial length, *HSAL* Haigis formula, single-optimized and AL-applied, *HSCL* Haigis formula, single-optimized and CMAL-applied, *HTAL* Haigis formula, triple-optimized and AL-applied, *HTCL* Haigis formula, triple-optimized and CMAL-applied, *EVO* Emmetropia Verifying Optical formula, *Hoffer QST* Hoffer Q/Savini/Taroni formula, *PEARL–DGS* Prediction Enhanced by Artificial Intelligence and output Linearization–Debellemanière Gatinel, and Saad.

The bolded values with asterisks (*) represent significant differences between the formulas compared.

While they exhibited statistically significant (P < 0.05, see Supplementary Table S1) hyperopic shifts, except for the Hoffer QST formula which resulted in myopia, their overall performances were generally better than those of the conventional axial length (AL)-applied Haigis formula (both single- and triple-optimized). However, it was not statistically significant by the root mean square error (RMSE) comparison (see Supplementary Table S2).

The newer formulas performed well in the medium eye subgroup. As this subgroup comprised > 90% of the entire population, accuracy in this subgroup was the main factor affecting overall performance (see Supplementary Table S3).

**Figure 1:**
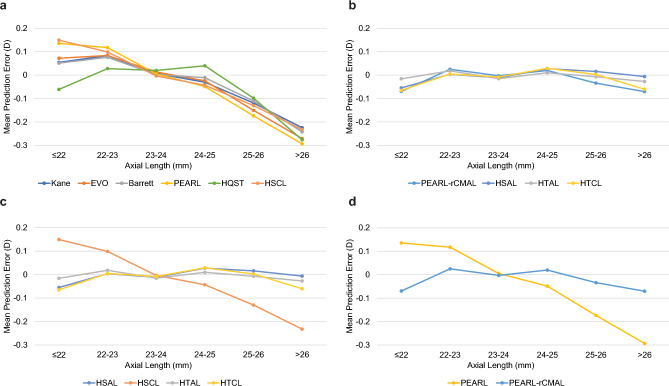
Mean prediction error plotted against the axial length groups of each formula. *AL* axial length, *CMAL* Cooke-modified axial length, *D* diopter, *HSAL* Haigis formula, single-optimized and AL-applied, *HSCL* Haigis formula, single-optimized and CMAL-applied, *HTAL* Haigis formula, triple-optimized and AL-applied, *HTCL* Haigis formula, triple-optimized and CMAL-applied, *HQST* Hoffer Q/Savini/Taroni formula, *PEARL-rCMAL* PEARL-DGS formula with reversed CMAL (= [AL + 0.05467 × lens thickness − 1.28353]/0.95855).

In the long eye subgroup, a marked difference was observed between the novel and Haigis formulas (Fig. 1, Table 4). All newer formulas showed a significant (P < 0.001, Table 4) myopic mean numerical prediction error (ME) ranging between − 0.2 D and − 0.3 D (Fig. 1a). In contrast, the Haigis formula (both single- and triple-optimized) had an ME close to zero (Fig. 1b). The myopic ME of the newer formulas increased the RMSE and absolute errors in this subgroup more than the older two-variable formulas in most cases, despite the SDs of the newer formulas being smaller. The RMSE comparison confirmed that the refractive errors of the new formulas were significantly higher than those of the Haigis (conventional AL-applied) formula (Table 5). The systemic deviation, not the random error, determined the performance and eventually affected the SD of the entire population.

**Table 4:** Prediction errors of each formula in the long axial length subgroup.

Formula	Long eyes (> 26 mm, N = 133)					
	ME	P-value	SD	RMSE	MedAE	MAE
PEARL	− 0.2933	1.3E−15*	0.3410	0.4471	0.2914	0.3581
HTCL	− 0.0585	0.4668	0.3344	0.3383	0.2158	0.2608
Kane	− 0.2245	**5.6E**−11*	0.3347	0.4019	0.2669	0.3212
EVO 2.0	− 0.2707	6.1E−14*	0.3409	0.4343	0.2958	0.3484
HTAL	− 0.0273	0.0731	0.3385	0.3383	0.2061	0.2585
HSAL	− 0.0062	0.9356	0.3400	0.3388	0.1991	0.2586
HSCL	− 0.2323	2.9E−11*	0.3433	0.4135	0.2652	0.3274
Barrett	− 0.2420	1.3E−13*	0.3377	0.4144	0.2738	0.3327
Hoffer QST	− 0.2702	9.5E−14*	0.3513	0.4421	0.3130	0.3596
Holladay 1	0.0987	0.0077*	0.3909	0.4018	0.2824	0.3271
Hoffer Q	0.1150	0.0005*	0.3913	0.4065	0.2490	0.3199
SRK/T	− 0.1222	0.0010*	0.3923	0.4095	0.3033	0.3372

The optimized constants for the formulas are: Hoffer Q pACD: 0.571, Holladay 1 SF: 1.859, SRK/T A constant: 119.076. For the Haigis formula, HSAL a0: 1.523, a1: 0.4, a2: 0.1, HTAL a0: 1.304, a1: 0.442, a2: 0.104, HSCL a0: 1.556, a1: 0.4, a2: 0.1, HTCL a0: 3.526, a1: 0.523, a2: 0, PEARL, A constant: 119.27; Kane, A constant: 119.16; EVO 2.0, A constant: 119.15; Barrett, A constant: 119.18; and Hoffer QST, pACD: 5.664.

*AL* axial length, *CMAL* Cooke-modified axial length, *HSAL* Haigis formula, single-optimized and AL-applied, *HSCL* Haigis formula, single-optimized and CMAL-applied, *HTAL* Haigis formula, triple-optimized and AL-applied, *HTCL* Haigis formula, triple-optimized and CMAL-applied, *MAE* mean absolute error, *ME* mean numerical prediction error, *MedAE* median absolute error, *RMSE* root mean square numerical error, *SD* standard deviation, *EVO* Emmetropia Verifying Optical formula, *Hoffer QST* Hoffer Q/Savini/Taroni formula, *PEARL–DGS* Prediction Enhanced by Artificial Intelligence and output Linearization–Debellemanière Gatinel, and Saad.

The bolded values with asterisks (*) in P-value column represent significant differences of the ME of each formula from 0.

**Table 5:** Statistical comparison of the root mean square error of the formulas for the long axial length subgroup with adjusted *P*-values (heteroscedastic test and Holm correction).

Formula	HTCL	Kane	EVO 2.0	PEARL	HTAL	HSAL	HSCL	Barrett
HTCL	–	–	–	–	–	–	–	–
Kane	0.0E+00*	–	–	–	–	–	–	–
EVO 2.0	0.0E+00*	0.0E+00*	–	–	–	–	–	–
PEARL	0.0E+00*	0.0100*	0.0811	–	–	–	–	–
HTAL	0.9530	0.0E+00*	0.0E+00*	0.0E+00*	–	–	–	–
HSAL	0.9530	0.0100*	0.0E+00*	0.0E+00*	0.8411	–	–	–
HSCL	0.0E+00*	0.7078	0.0E+00*	0.0E+00*	0.0E+00*	0.0100*	–	–
Barrett	0.0E+00*	0.4134	0.0100*	0.0100*	0.0E+00*	0.0100*	0.9530	–
Hoffer QST	0.0E+00*	0.0E+00**	0.9530	0.9530	0.0E+00*	0.0E+00*	0.0E+00*	0.0E+00*

*AL* axial length, *CMAL* Cooke-modified axial length, *HSAL* Haigis formula, single-optimized and AL-applied, *HSCL* Haigis formula, single-optimized and CMAL-applied, *HTAL* Haigis formula, triple-optimized and AL-applied, *HTCL* Haigis formula, triple-optimized and CMAL-applied, *EVO* Emmetropia Verifying Optical formula, *Hoffer QST* Hoffer Q/Savini/Taroni formula, *PEARL–DGS* Prediction Enhanced by Artificial Intelligence and output Linearization–Debellemanière, Gatinel, and Saad.

The bolded values with asterisks (*) represent significant differences between the formulas compared.

The experimentally Cooke-modified AL (CMAL)-applied, single-optimized Haigis formula showed a similar myopic shift in long eyes (Fig. 1c). The correlation between the newer and Haigis formulas’ prediction error increased with CMAL replacement (Table 6).

**Table 6:** Coefficients of determination (R^2^ values) between prediction errors of formulas.

Formula	HSAL	HSCL
PEARL	0.6894	**0.8503**
Barrett	0.7027	**0.7638**
EVO 2.0	0.7095	**0.8076**
Kane	0.7159	**0.8157**
Hoffer QST	0.8263	**0.8272**
PEARL-rCMAL	**0.8429**	0.81

Formula	PEARL	PEARL-rCMAL
Barrett	**0.8527**	0.8501
EVO 2.0	**0.9503**	0.9106
Kane	**0.9435**	0.9031
Hoffer QST	0.7582	**0.8152**

*AL* axial length, *CMAL* Cooke-modified axial length, *HSAL* Haigis formula, single-optimized and AL-applied, *HSCL* Haigis formula, single-optimized and CMAL-applied, *PEARL-rCMAL* PEARL-DGS formula with reversed CMAL (= ([AL + 0.05467 × lens thickness − 1.28353]/0.95855), *R* Pearson correlation coefficient, *EVO* Emmetropia Verifying Optical formula, *Hoffer QST* Hoffer Q/Savini/Taroni formula, *PEARL–DGS* Prediction Enhanced by Artificial Intelligence and output Linearization–Debellemanière, Gatinel, and Saad, *HTAL* Haigis formula, triple-optimized and AL-applied, *HTCL* Haigis formula, triple-optimized and CMAL-applied, *HQST* Hoffer Q/Savini/Taroni formula.

The bolded values represent higher Coefficients of determination (R^2^ values).

Additionally, with CMAL application and triple optimization, the SD of the Haigis formula markedly decreased, even significantly smaller than that of the Barrett and Hoffer QST formulas (Tables 2, 3).

Another experiment with the PEARL-DGS formula, replacing the AL with the reversed CMAL (AL + 0.05467 × lens thickness [LT] − 1.23853)/0.95855), calculated using the conventional AL instead of the CMAL in its inner algorithm^15^, showed that the myopic shift substantially decreased in the long AL subgroup (Fig. 1d), and the overall SD decreased (see Supplementary Table S4) significantly (P < 0.001). The correlations between the prediction errors of the PEARL and EVO, as well as between PEARL and Kane formulas decreased with the reversed CMAL (Table 6). The comparison between the average values of conventional AL, CMAL, and reversed CMAL in AL subgroups are described (see Supplementary Table S5).

**Figure 2:**
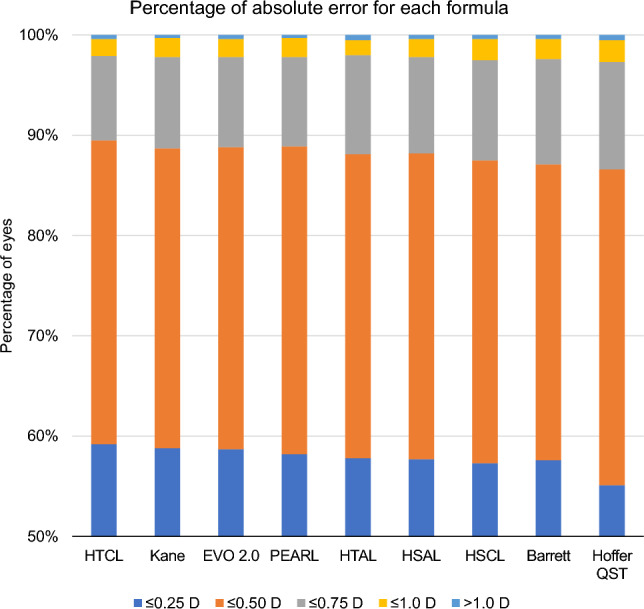
Percentage of absolute errors of each formula. *AL* axial length, *CMAL* Cooke-modified axial length, *D* diopter, *HSAL* Haigis formula, single-optimized and AL-applied, *HSCL* Haigis formula, single-optimized and CMAL-applied, *HTAL* Haigis formula, triple-optimized and AL-applied, *HTCL* Haigis formula, triple-optimized and CMAL-applied.

The Cochran’s Q test revealed significant differences in the percentage of cases within 0.25, 0.50, and 0.75 D, but not within the 1.0 D range of absolute errors, as all formulas recorded > 99.4% within the 1.0 D range. All the newer formulas showed > 86.6% within the 0.50 D range (Fig. 2). However, they did not outperform the Haigis formula (both single- and triple-optimized) (see Supplementary Table S5).

The separate results of the non-toric and toric IOL subgroups exhibited no significant differences compared to the overall analysis, including the newer formulas’ myopic tendencies in long eyes (see Supplementary Tables S6–S8).

## Discussion

To our knowledge, this report comprises the largest study population for the TFNT IOL and adheres to the generally accepted guidelines^22–24^. The use of high-quality data from uniform clinical settings, including a single IOL model and the same biometer throughout the study, is another strength of this study. Additionally, we compared the accuracy of the novel IOL formulas. The superiority of the newer formulas to the older two-variable formulas, as previously demonstrated with monofocal IOLs^2,18,21^ is well reproduced, herein.

The performance of the minimally structured Haigis formula was comparable to that of the more sophisticated newer formulas; none of them significantly outperformed the Haigis formula. Interestingly, although there was no notable difference between the single-optimized, triple-optimized AL-applied, and single-optimized CMAL-applied Haigis formulas, the accuracy of the triple-optimized CMAL-applied version improved significantly (P < 0.001, Table 3). The triple-optimized CMAL-applied Haigis formula was significantly superior to the Barrett and Hoffer QST formulas (P < 0.001, Table 3). A noticeable change was also observed in the optimized constant triplets with the CMAL (3.526/0.523/0) compared with those with AL (1.304/0.442/0.104), which demonstrated little difference from the default values (a1: 0.4, a2: 0.1). The nullification of the a2 constant is noteworthy, indicating that the CMAL had no influence on the ELP prediction.

While our results are divergent from previous large population studies that demonstrated the superiority of the newer formulas over the Haigis formula^2,18,20,21^, they align well with the fundamental principle that formula accuracy comparison results can vary significantly depending on factors such as the test population characteristics, constant optimization, and clinical settings^17,22^. In our population, the alleged exceptional adjustability of the triplet-constant Haigis formula^15,17^ enabled it to demonstrate comparability to the more complex but less flexible single-constant novel formulas. Depending on the clinical setting, investing extra time and effort in the undisclosed working of the online calculators may not guarantee more advantages than actually optimizing the Haigis formula to its own population and relying on it.

The most distinct outcome of this study was that all novel formulas showed statistically significant hyperopic results in short eyes (except for the Hoffer QST formula), and more pronounced myopic outcomes in long eyes (Fig. 1a), which contradicts the findings of previous reports^26–28^. Contrarily, the Haigis formula that used the conventional AL exhibited consistent performance throughout the entire AL (Fig. 1b). The IOLMaster 700 follows the AL calculation of its predecessors^13^. It tends to underestimate the AL in shorter eyes and overestimate it in longer eyes^13^, consequently resulting in myopic errors in shorter eyes and hyperopic errors in longer eyes^13,26–28^. To compensate for this, the CMAL was developed and is considered to better resemble the actual AL^26^. The PEARL-DGS formula is known to incorporate the CMAL in its algorithm^15^; this was ensured after a recent update (confirmed by the author through personal communication). Moreover, the Hoffer QST formula incorporated a customized AL adjustment algorithm developed using machine learning methods^25^.

As there was no way to further investigate the underlying causes of the unpublished newer formulas’ myopic shifts in the long eyes, we experimentally applied the CMAL to the Haigis formula and single optimized, expecting that this experimental approach would provide insights into the workings of the newer formulas (both share the same optical principles, single constant, and AL modification). The single-optimized CMAL-applied Haigis formula revealed similar hyperopic shifts in short eyes and myopic shifts in long eyes (Fig. 1c), and the overall SD remained nearly unchanged (Table 2). The correlation between the errors of the Haigis and newer formulas increased after applying the CMAL to the Haigis formula with single optimization (Table 6). We also experimented with the online PEARL-DGS formula calculator, entering a reversed CMAL, calculating with the conventional AL instead of the CMAL in its inner algorithm^15^. The results showed that the myopic shift substantially decreased in the long AL subgroup (Fig. 1d), and the overall SD decreased significantly (P < 0.001, see Supplementary Table S4). The correlation between the errors of the PEARL and EVO, as well as between the PEARL and Kane formulas decreased with the reversed CMAL (Table 6).

Based on our results, we speculate that other three novel formulas (Barrett, EVO, and Kane) also use some AL modification algorithms more or less similar to the CMAL in their algorithms, which seem to overcorrect, yielding statistically significant hyperopic errors in short eyes and more pronounced myopic errors in the long eyes of our population. Future studies should further investigate the myopic results in the long AL range.

Another remarkable strength of this study was the clinical relevance of the results. Without awareness of this myopic deviation of the newer formulas in the long AL range, surgeons might deliberately aim for myopia in selecting IOL power, as in a monofocal IOL implantation, or in an attempt to compensate for the well-known hyperopic shifts^26–28^. For instance, in a normal distribution of postoperative refraction error when targeting emmetropia with an ME of − 0.28 D and an SD of 0.35 D, aiming for a mean refraction target of − 0.21 D would result in a proportion of 7.2% exceeding − 1.0 D, which corresponds to an approximately three-step difference in IOL power in long eyes. This extent of residual myopia, considering the low tolerance of multifocal IOL designs for myopia^10^, may potentially compromise spectacle independence and necessitate secondary interventions, such as IOL exchange or laser corneal surgeries. In case of a more myopic target or bigger SD, these suboptimal proportions would escalate. Moreover, as the population size increases, the clinical relevance of these findings would become more pronounced^19^. The less apparent scale of this pattern, compared to the well-known hyperopia in older formulas^26–28^, might have allowed them to be left undetected, thereby insidiously affecting the refractive outcomes.

A limitation of this study is the inclusion of eyes that underwent IOL exchange. To relieve patients’ early preoperative discomfort, IOL exchange was performed within 3 months postoperatively before strong adhesion between the IOL and the lens capsule was established. Despite these eyes having no chance of being evaluated during the same period as others, excluding them would have resulted in a positive bias. Therefore, we included them, and the refractive results obtained at least 3 months after IOL exchange were used instead of the early post-cataract surgery outcomes to best meet the standards of this study.

In conclusion, although the new formulas performed well in Korean patients with multifocal TFNT IOL implantation, they did not show significant superiority over the Haigis formula using conventional AL, mainly because of substantial myopic errors in the long AL subgroup. The Haigis formula with CMAL application and single optimization showed similar myopic shifts in long eyes, while triple optimization yielded a significantly smaller SD than some of the newer formulas. Considering the potential clinical consequences when combined with conventional myopic targeting, surgeons implanting the same IOL in East Asian patients need to be aware of the newer formulas’ potential myopic shifts in long eyes and take them into consideration when selecting IOL power.

## Methods

This study conformed to the tenets of the Declaration of Helsinki and was approved by the Korean Public Institutional Review Board. Due to the anonymized data extraction and analysis, the requirement for informed consent was waived by the Korean Public Institutional Review Board.

We performed a retrospective chart review of consecutive patients who underwent cataract surgery between January 2020 and April 2022 at the Miracle eye clinic, Seoul, Korea. Eyes with uneventful in-the-bag implantation of the AcrySof TFNT IOL (including toric versions; Alcon Laboratories, Fort Worth, TX) were selected. The specifications of the studied IOL have been described in the literature^29^.

All patients were evaluated using IOLMaster 700 (software versions 1.88 to 1.90; Carl Zeiss Meditec AG, Jena, Germany) to obtain the following parameters: AL, conventional keratometry, total keratometry (TK), central corneal thickness (CCT), anterior chamber depth (measured from the corneal epithelium to the lens), LT, and horizontal corneal diameter (also known as white-to-white)^22^. The TK value was used to represent the corneal power in practice and in this study. The selection of IOL power in practice was based on the biometer printout, which provided results of four formulas using default constants: SRK/T (A-constant: 119.1), Hoffer Q (pACD: 5.61), Haigis (a0: 1.390, a1: 0.4, a2: 0.1), and Barrett (LF: 1.94, equivalent to A-constant 119.1 on the online calculator). Previous large population studies^2,18,21^ have consistently shown that the Barrett formula is the most accurate among these. Therefore, the recommendation of the Barrett formula was primarily followed, especially in situations where there were discrepancies among the recommendations.

Four experienced surgeons performed all surgeries using the standard technique. Candidates for multifocal IOL implantation were strictly screened^3,7,8^. Surgeons strongly discouraged performing implantation on patients with any significant visual impairment due to ocular pathology other than cataracts. Patients with these unfavorable conditions, in whom the procedure was not indicated and who underwent implantation at their own request, were eventually excluded from the analysis. Eyes that had undergone additional previous or postoperative ocular surgery that may affect refractive status and eyes with intra- or postoperative complications were excluded. As the studied IOL has a power range of 6.0–34.0 dioptres (D) and a toric range of 1.0–3.75 D, extremely long (> 30 mm) or short eyes (< 20 mm) and eyes with severe corneal astigmatism (> 3.5 D) were automatically excluded.

Final postoperative refraction was evaluated by in-house optometrists using an automated refractometer (RK-F2, Canon, Tokyo, Japan) and confirmed subjectively by non-cycloplegic manifest refraction, using a 4 m lane and a − 0.25 D adjustment, at least 3 months after surgery^22,23^. Twenty-six eyes (0.8%) underwent IOL exchange with the same IOL model at different powers or toricities to reduce postoperative refractive errors, mostly within 1 month postoperatively. For these eyes, the final refractive results were obtained at least 3 months after the IOL exchange. Predicted refractions were calculated from the final implanted IOL power and biometric data before cataract surgery. Of the 5973 eyes that met the study criteria, by using the RAND() function in Excel spreadsheets (Microsoft, Redmond, WA), one eye from each patient was randomly selected^22–24^.

The Holladay 1^30^, Hoffer Q^31^, SRK/T^32^, and Haigis^33^ formulas were programmed into Excel spreadsheets.

The online calculators of the Barrett Universal II^34^, EVO 2.0^35^, Kane^36^, and PEARL-DGS^37^ formulas were accessed using a robotic process automation software (UIPath Studio, Uipath, New York, NY). If the implanted IOL power was not within the range suggested by each formula, as in cases of IOL exchange, the predicted refraction was obtained by entering a different target refraction other than 0. The Hoffer QST formula’s^25^ dedicated research section^38^ was used for optimized calculation (optimized pACD: 5.664).

The Hill-RBF formula 3.0^39^ was not included because its online calculator refused automated access, and manual data transcription was not an alternative option because of the large data size. Instead, to obtain results from pure artificial intelligence (AI) based formula, the Nallasamy formula^40^ was included for the initial calculation. Another pure AI-based formula, the Karmona formula^41^, was not considered due to its relatively smaller training population size.

The Excel results were verified using biometer printouts. The results of the online calculators were verified by manually entering data from 31 randomly selected eyes (1% of all data).

Constant optimization for the Excel-programmed single constant formulas (Hoffer Q pACD: 0.571, Holladay 1 SF: 1.859, SRK/T A constant: 119.076, Haigis a0: 1.523) was conducted using Data/What If Analysis/Goal Seek function^22,23^. For triple constant optimization of the Haigis formula (a0: 1.304/a1: 0.442/a2: 0.104), multiple linear regression analysis was conducted using the Python programming language (Python Software Foundation, Wilmington, DE)^22,23^.

For the newer formulas, except for the Hoffer QST formula, in the early stage of this study, neither independent constant optimization nor constants from the User Group for Laser Interference Biometry were available^18,42^. Therefore, a manufacturer-provided constant of 119.1 (also the default constant of the biometer) was entered into the online calculators and their mean prediction errors were adjusted to zero by subtracting the mean prediction error from each eye’s refractive error^24,43^.

Initially, the optimized constant (119.27) and corresponding results for the PEARL–DGS formula had been obtained from the author of the formula (G. Debellemanière, personal communication).

Later, further attempts were made to optimize the constants of the unpublished formulas through recent publications^44,45^. Initially, the earlier published method^44^ was tested multiple times. However, this method failed to contribute to constant optimization. The program provided results upon completion of calculations, but errors unexpectedly occurred at various iteration points during each trial, leading the program to halt without leaving any intermediate results. These results could have been valuable as starting points for subsequent calculations, reducing the overall completion time. However, as the program did not provide such functionality, every calculation had to be restarted from the beginning and subsequently halted, resulting in no obtained results. This was possibly attributed to the large sample size involved. Another recently published method^45^, which involves a mathematical equation, calculates the mean ELP difference by taking into account the mean keratometric value, mean IOL power, and ME for a given A constant. This mean ELP difference is then used to iteratively adjust the A constant until the ME is close to 0. By utilizing this approach, constant optimizations for the Barrett (119.18), EVO 2.0 (119.15), and Kane (119.16) formulas were completed.

Since the Nallasamy formula does not rely on ELP prediction for IOL power calculation^19^, it does not utilize an IOL constant either. As a result, there was no method to obtain optimized results from that formula. Therefore, to ensure a fair comparison, the Nallasamy formula was excluded from further analysis.

For five eyes with CCT > 650 µm (range 651 − 685 µm), a value of 650 µm was entered into the Kane formula, as this was its upper limit.

After the completion of this paper, a substantial discrepancy was discovered in the online calculator of the PEARL-DGS formula compared to the original version used in this study. The white-to-white (WTW) value was found to be omitted from the data input page. Furthermore, the prediction value obtained from the same input data had been altered (see Supplementary Fig. S1), leading to a significant impact on the overall results and conclusions. To maintain the clinical relevance of this study, the results of the PEARL-DGS formula were recalculated using the updated version of the online calculator. The optimized constant for the updated PEARL-DGS formula was calculated using the same methodology^45^ employed for the Barrett, EVO 2.0, and Kane formula, and was confirmed to be unchanged (119.27). Subsequently, all the recalculated comparisons replaced the previous results, and the manuscript was revised accordingly.

All eyes were categorized as short (AL < 22 mm), medium (22 ≤ AL ≤ 26 mm), and long (AL > 26 mm) for subgroup analysis. The prediction error was calculated by subtracting the predicted refraction from the actual postoperative refraction^24^. The ME, SD, and RMSE were calculated from the numerical errors. The median absolute error and mean absolute error were calculated after conversion to absolute errors. The percentages of eyes within absolute errors of 0.25, 0.5, 0.75, and 1.0 D were assessed^22^.

The normality of numerical errors was assessed using the Shapiro–Wilk test. The SD of each formula was compared using the heteroscedastic test recommended by Holladay^46^. For subgroup comparisons, the RMSE of each formula was compared using the heteroscedastic test^47^. Percentages within the absolute error ranges were assessed using Cochran’s Q and subsequent McNemar tests. Holm correction was performed for post hoc analysis^46^. The Pearson correlation coefficients between the prediction errors of Haigis, and other newer formulas were calculated. In addition, to investigate the impact of the AL modification on the results of the Haigis formula, as conducted in other studies^48,49^, the CMAL was experimentally implemented to the Haigis formula; thereafter, single (a0: 1.556) and triple optimizations (a0: 3.526/a1: 0.523/a2: 0) were performed. Conversely, to evaluate the performance of newer formulas without the AL modification, an experiment to replace the AL with the reversed CMAL was performed to the PEARL-DGS formula’s online calculator with constant optimization (119.23).

Statistical analyses were performed using Holladay’s software package^47^ based on R software (version 3.3.3; R Foundation, Vienna, Austria). A Holm-adjusted *P*-value < 0.05 was considered statistically significant.

### Supplementary Information

Supplementary Information.

## Data Availability

The datasets generated and/or analyzed during the current study are not publicly available but are available from the corresponding author on reasonable request.
