# Supplementary material for: Comparison of the formula accuracy for calculating multifocal intraocular lens power: a single center retrospective study in Korean patients
Source: Sci Rep. 2024 Feb 23;14:4462. doi: 10.1038/s41598-024-54889-x (PMC10891126; doi:10.1038/s41598-024-54889-x)
Supplement: Supplementary file 1 — Supplementary Information. [file 41598_2024_54889_MOESM1_ESM.docx]

**Supplementary Information**

**Comparison of the formula accuracy for calculating multifocal intraocular lens power in Asian patients**

Jinchul Kim, Joonsung Park, Yoonjung Jo

Supplementary Table S1. Prediction errors of each formula in the short axial length subgroup

|  | **Short eyes** (<22 mm, N=134) | | | | | |
| --- | --- | --- | --- | --- | --- | --- |
| Formula | ME | P-value | SD | RMSE | MedAE | MAE |
| PEARL | 0.1388 | 1.8E-08* | 0.2681 | 0.3010 | 0.1724 | 0.2301 |
| HTCL | -0.0633 | 0.0216* | 0.3153 | 0.3204 | 0.1887 | 0.2381 |
| Kane | 0.0576 | 0.0177* | 0.2773 | 0.2822 | 0.1719 | 0.2163 |
| EVO 2.0 | 0.0757 | 0.0016* | 0.2723 | 0.2818 | 0.1742 | 0.2174 |
| HTAL | -0.0143 | 0.6100 | 0.3234 | 0.3225 | 0.1915 | 0.2436 |
| HSAL | -0.0533 | 0.0591 | 0.3241 | 0.3272 | 0.1840 | 0.2502 |
| HSCL | 0.1515 | 6.3E-08* | 0.3058 | 0.3402 | 0.1954 | 0.2635 |
| Barrett | 0.0546 | 0.0486* | 0.3179 | 0.3214 | 0.2058 | 0.2524 |
| Hoffer QST | -0.0582 | 0.0365* | 0.3190 | 0.3230 | 0.2200 | 0.2598 |
| Holladay 1 | -0.0171 | 0.5568 | 0.3351 | 0.3343 | 0.2371 | 0.2703 |
| Hoffer Q | -0.2021 | 3.3E-10* | 0.3444 | 0.3982 | 0.2709 | 0.3167 |
| SRK/T | -0.0064 | 0.8464 | 0.3811 | 0.3797 | 0.2490 | 0.3091 |

The optimized constants for the formulas are: Hoffer Q pACD: 5.71, Holladay 1 SF: 1.859, SRK/T A constant: 119.076. For the Haigis formula, HSAL a0: 1.523, a1: 0.4, a2: 0.1, HTAL a0: 1.304, a1: 0.442, a2: 0.104, HSCL a0: 1.556, a1: 0.4, a2: 0.1, HTCL a0: 3.526, a1: 0.523, a2: 0, PEARL, A constant: 119.27; Kane, A constant: 119.16; EVO 2.0, A constant: 119.15; Barrett, A constant: 119.18; and Hoffer QST, pACD: 5.664.

The asterisks (*) in P-value column represent significant differences of the

ME of each formula from 0.

AL, axial length; CMAL, Cooke-modified axial length; HSAL, Haigis formula, single-optimized and AL-applied; HSCL, Haigis formula, single-optimized and CMAL-applied; HTAL, Haigis formula, triple-optimized and AL-applied; HTCL, Haigis formula, triple-optimized and CMAL-applied; MAE, mean absolute error; ME, mean numerical prediction error; MedAE, median absolute error; RMSE, root mean square numerical error; SD, standard deviation; EVO, Emmetropia Verifying Optical formula; Hoffer QST, Hoffer Q/Savini/Taroni formula; MAE, mean absolute error; ME, mean numerical prediction error; MedAE, median absolute error; PEARL–DGS, Prediction Enhanced by Artificial Intelligence and output Linearization–Debellemanière, Gatinel, and Saad; RMSE, root mean square numerical error; SD, standard deviation.

Supplementary Table S2. Root mean square error comparisons in the short axial length subgroup with adjusted *P*-values (heteroscedastic test and Holm correction)

| Formula | HTCL | Kane | EVO 2.0 | PEARL | HTAL | HSAL | HSCL | Barrett |
| --- | --- | --- | --- | --- | --- | --- | --- | --- |
| HTCL | - | - | - | - | - | - | - | - |
| Kane | 0.9720 | - | - | - | - | - | - | - |
| EVO 2.0 | 0.9720 | 0.9720 | - | - | - | - | - | - |
| PEARL | 0.9720 | 0.9720 | 0.8809 | - | - | - | - | - |
| HTAL | 0.97200 | 0.9362 | 0.9362 | 0.9720 | - | - | - | - |
| HSAL | 0.9720 | 0.9362 | 0.9362 | 0.7488 | 0.9720 | - | - | - |
| HSCL | 0.9720 | 0.E+00* | 0.E+00* | 0.0013* | 0.9720 | 0.9720 | - | - |
| Barrett | 0.0003* | 0.E+00* | 0.0991 | 0.9720 | 0.4920 | 0.9720 | 0.3904 | - |
| Hoffer QST | 0.9720 | 0.6887 | 0.6727 | 0.9720 | 0.9720 | 0.9720 | 0.9720 | 0.9720 |

The asterisks (*) represent significant differences between the formulas

compared.

AL, axial length; CMAL, Cooke-modified axial length; HSAL, Haigis formula, single-optimized and AL-applied; HSCL, Haigis formula, single-optimized and CMAL-applied;

HTAL, Haigis formula, triple-optimized and AL-applied; HTCL, Haigis formula, triple-optimized and CMAL-applied; EVO, Emmetropia Verifying Optical formula; Hoffer QST, Hoffer Q/Savini/Taroni formula; PEARL–DGS, Prediction Enhanced by Artificial Intelligence and output Linearization–Debellemanière, Gatinel, and Saad.

Supplementary Table S3. Prediction errors of each formula in the medium axial length subgroup

| **Medium eyes** (22–26 mm, N=2833) | | | | | |
| --- | --- | --- | --- | --- | --- |
| Formula | ME | SD | RMSE | MedAE | MAE |
| PEARL | 0.0072 | 0.3107 | 0.3108 | 0.2007 | 0.2438 |
| HTCL | 0.0041 | 0.3117 | 0.3117 | 0.2006 | 0.2453 |
| Kane | 0.0078 | 0.3137 | 0.3138 | 0.2019 | 0.246 |
| EVO 2.0 | 0.0091 | 0.3120 | 0.3121 | 0.2008 | 0.2451 |
| HTAL | -0.0002 | 0.3213 | 0.3212 | 0.2146 | 0.2534 |
| HSAL | 0.0035 | 0.3215 | 0.3215 | 0.2129 | 0.2542 |
| HSCL | 0.0038 | 0.3206 | 0.3206 | 0.2089 | 0.2524 |
| Barrett | 0.0088 | 0.3238 | 0.3238 | 0.2112 | 0.2544 |
| Hoffer QST | 0.0154 | 0.3302 | 0.3305 | 0.2190 | 0.2618 |
| Holladay 1 | -0.0038 | 0.3486 | 0.3486 | 0.2343 | 0.2763 |
| Hoffer Q | 0.0042 | 0.3484 | 0.3484 | 0.2346 | 0.2769 |
| SRK/T | 0.0056 | 0.3897 | 0.3897 | 0.2548 | 0.3077 |

The optimized constants for the formulas are: Hoffer Q pACD: 5.71, Holladay 1 SF: 1.859, SRK/T A constant: 119.076. For the Haigis formula, HSAL a0: 1.523, a1: 0.4, a2: 0.1, HTAL a0: 1.304, a1: 0.442, a2: 0.104, HSCL a0: 1.556, a1: 0.4, a2: 0.1, HTCL a0: 3.526, a1: 0.523, a2: 0, PEARL, A constant: 119.27; Kane, A constant: 119.16; EVO 2.0, A constant: 119.15; Barrett, A constant: 119.18; and Hoffer QST, pACD: 5.664.

AL, axial length; CMAL, Cooke-modified axial length; HSAL, Haigis formula, single-optimized and AL-applied; HSCL, Haigis formula, single-optimized and CMAL-applied; HTAL, Haigis formula, triple-optimized and AL-applied; HTCL, Haigis formula, triple-optimized and CMAL-applied; MAE, mean absolute error; ME, mean numerical prediction error; MedAE, median absolute error; RMSE, root mean square numerical error; SD, standard deviation; EVO, Emmetropia Verifying Optical formula; Hoffer QST, Hoffer Q/Savini/Taroni formula; MAE, mean absolute error; ME, mean numerical prediction error; MedAE, median absolute error; PEARL–DGS, Prediction Enhanced by Artificial Intelligence and output Linearization–Debellemanière, Gatinel, and Saad; RMSE, root mean square numerical error; SD, standard deviation.

Supplementary Table S4. Prediction errors of the conventional AL-applied, and reversed CMAL-applied PEARL-DGS formulas

| Whole Group (N=3100) | | | | |
| --- | --- | --- | --- | --- |
| Formula | ME | SD | MedAE | MAE |
| PEARL | -1.6E-06 | 0.3175 | 0.2040 | 0.2302 |
| PEARL-rCMAL | 2.5E-05 | 0.3023 | 0.1921 | 0.2199 |

| Short Eyes (<22 mm, N=134) | | | | | |
| --- | --- | --- | --- | --- | --- |
| Formula | ME | SD | RMSE | MedAE | MAE |
| PEARL | 0.1388 | 0.2681 | 0.3010 | 0.1724 | 0.2302 |
| PEARL-rCMAL | -0.0667 | 0.2773 | 0.2842 | 0.1780 | 0.2199 |

| Medium Eyes (22–26 mm, N=2833) | | | | | |
| --- | --- | --- | --- | --- | --- |
| Formula | ME | SD | RMSE | MedAE | MAE |
| PEARL | 0.0072 | 0.3107 | 0.3107 | 0.2007 | 0.2437 |
| PEARL-rCMAL | 0.0065 | 0.3010 | 0.3010 | 0.1909 | 0.2356 |

| Long Eyes (>26 mm, N=133) | | | | | |
| --- | --- | --- | --- | --- | --- |
| Formula | ME | SD | RMSE | MedAE | MAE |
| PEARL | -0.2933 | 0.3410 | 0.4488 | 0.2924 | 0.3605 |
| PEARL-rCMAL | -0.0704 | 0.3377 | 0.3437 | 0.2381 | 0.2632 |

CMAL, Cooke-modified axial length; MAE = mean absolute error; ME = mean numerical prediction error; MedAE = median absolute error; PEARL-rCMAL = PEARL-DGS formula with reversed CMAL (= ([AL + 0.05467 × lens thickness – 1.28353]/ 0.95855); RMSE = root mean square error; SD = standard deviation.

The optimized constants for the formulas are: PEARL, A constant: 119.27; PEARL-rCMAL, A constant: 119.23.

Supplementary Table S5. Comparison between the average values of conventional AL, CMAL, and reversed CMAL in AL subgroups.

| **AL** | Whole Group (N=3100) | Short Eyes  (<22 mm, N=134) | Medium Eyes (22–26 mm, N=2833) | Long Eyes  (>26 mm, N=133) |
| --- | --- | --- | --- | --- |
| CMAL (mm) | 23.69 | 21.78 | 23.65 | 26.66 |
| Conventional AL (mm) | 23.68 | 21.69 | 23.63 | 26.77 |
| reversed CMAL (mm) | 23.67 | 21.60 | 23.62 | 26.88 |

AL, axial length; CMAL, Cooke-modified axial length; reversed CMAL (= ([AL + 0.05467 × lens thickness – 1.28353]/ 0.95855).

Supplementary Table S6. Statistical comparisons of the percentage within 0.50 D for the whole group with adjusted *P*-values (Cochran’s Q test, subsequent pairwise McNemar test, and Holm correction)

| Formula | HTCL | Kane | EVO 2.0 | PEARL | HTAL | HSAL | HSCL | Barrett |
| --- | --- | --- | --- | --- | --- | --- | --- | --- |
| HTCL | - | - | - | - | - | - | - | - |
| Kane | 1.0E+00 | - | - | - | - | - | - | - |
| EVO 2.0 | 1.0E+00 | 1.0E+00 | - | - | - | - | - | - |
| PEARL | 1.0E+00 | 1.0E+00 | 1.0E+00 | - | - | - | - | - |
| HTAL | 2.0E-05* | 1.0E+00 | 1.0E+00 | 1.0E+00 | - | - | - | - |
| HSAL | 0.0003* | 1.0E+00 | 1.0E+00 | 1.0E+00 | 1.0E+00 | - | - | - |
| HSCL | 7.5E-05* | 0.3841 | 0.2403 | 0.0632 | 1.0E+00 | 1.0E+00 | - | - |
| Barrett | 0.0005* | 0.0134* | 0.0001* | 0.0049* | 1.0E+00 | 1.0E+00 | 1.0E+00 | - |
| Hoffer QST | 8.4E-07* | 0.0026* | 0.0005* | 0.0013* | 0.1018 | 0.0408* | 1.0E+00 | 1.0E+00 |

The asterisks (*) represent significant differences between the formulas

compared.

AL, axial length; CMAL, Cooke-modified axial length; HSAL, Haigis formula, single-optimized and AL-applied; HSCL, Haigis formula, single-optimized and CMAL-applied; HTAL, Haigis formula, triple-optimized and AL-applied; HTCL, Haigis formula, triple-optimized and CMAL-applied; EVO, Emmetropia Verifying Optical formula; Hoffer QST, Hoffer Q/Savini/Taroni formula; PEARL–DGS, Prediction Enhanced by Artificial Intelligence and output Linearization–Debellemanière, Gatinel, and Saad.

Supplementary Table S7. Prediction errors of each formula in non-toric versus toric cases

1. Standard deviation of each formula in non-toric versus toric cases

| Formula | Whole (N=3100) | NT (N=975) | T (N=2125) |
| --- | --- | --- | --- |
| PEARL | 0.3175 | 0.3125 | 0.3197 |
| HTCL | 0.3133 | 0.3181 | 0.3100 |
| Kane | 0.3168 | 0.3110 | 0.3194 |
| EVO 2.0 | 0.3171 | 0.3117 | 0.3196 |
| HTAL | 0.3221 | 0.3261 | 0.3191 |
| HSAL | 0.3226 | 0.3273 | 0.3192 |
| HSCL | 0.3260 | 0.3259 | 0.3254 |
| Barrett | 0.3282 | 0.3227 | 0.3307 |
| Hoffer QST | 0.3358 | 0.3351 | 0.3360 |
| Holladay 1 | 0.3505 | 0.3483 | 0.3515 |
| Hoffer Q | 0.3534 | 0.3594 | 0.3495 |
| SRK/T | 0.3902 | 0.3780 | 0.3957 |

1. Mean numerical prediction error of each formula in long eyes (AL > 26 mm) in non-toric versus toric cases

| Formula | Whole (N=133) | NT (N=26) | T (N=107) |
| --- | --- | --- | --- |
| PEARL | -0.2933 | -0.2928 | -0.2934 |
| HTCL | -0.0603 | -0.0619 | -0.0599 |
| Kane | -0.2245 | -0.2243 | -0.2246 |
| EVO 2.0 | -0.2707 | -0.2635 | -0.2725 |
| HTAL | -0.0273 | -0.0362 | -0.0251 |
| HSAL | -0.0062 | -0.0122 | -0.0047 |
| HSCL | -0.2323 | -0.2361 | -0.2314 |
| Barrett | -0.2420 | -0.2330 | -0.2442 |
| Hoffer QST | -0.2702 | -0.2825 | -0.2744 |
| Holladay 1 | 0.0987 | 0.1212 | 0.0932 |
| Hoffer Q | 0.1150 | 0.1025 | 0.1181 |
| SRK/T | -0.1221 | -0.0781 | -0.1328 |

The optimized constants for the formulas are: Hoffer Q pACD: 5.71, Holladay 1 SF: 1.859, SRK/T A constant: 119.076. For the Haigis formula, HSAL a0: 1c.523, a1: 0.4, a2: 0.1, HTAL a0: 1.304, a1: 0.442, a2: 0.104, HSCL a0: 1.556, a1: 0.4, a2: 0.1, HTCL a0: 3.526, a1: 0.523, a2: 0, PEARL, A constant: 119.27; Kane A, constant: 119.16; EVO 2.0, A constant: 119.15; Barrett, A constant: 119.18; and Hoffer QST, pACD: 5.664.

AL, axial length; CMAL, Cooke-modified axial length; HSAL, Haigis formula, single-optimized and AL-applied; HSCL, Haigis formula, single-optimized and CMAL-applied; HTAL, Haigis formula, triple-optimized and AL-applied; HTCL, Haigis formula, triple-optimized and CMAL-applied; IOL, intraocular lens; NT, non-toric IOL; T, toric IOL;

EVO, Emmetropia Verifying Optical formula; Hoffer QST, Hoffer Q/Savini/Taroni formula; PEARL–DGS, Prediction Enhanced by Artificial Intelligence and output Linearization–Debellemanière, Gatinel, and Saad.

Supplementary Table S8. Statistical comparison of formulas in non-toric and toric cases

1. Comparisons of the standard deviation for the non-toric subgroup with adjusted *P*-values (heteroscedastic test and Holm correction)

| Formula | HTCL | Kane | EVO 2.0 | PEARL | HTAL | HSAL | HSCL | Barrett |
| --- | --- | --- | --- | --- | --- | --- | --- | --- |
| HTCL | - | - | - | - | - | - | - | - |
| Kane | 0.9434 | - | - | - | - | - | - | - |
| EVO 2.0 | 0.9434 | 0.9434 | - | - | - | - | - | - |
| PEARL | 0.9434 | 0.9434 | 0.9434 | - | - | - | - | - |
| HTAL | 2.6E-08* | 0.1114 | 0.1502 | 0.2622 | - | - | - | - |
| HSAL | 4.8E-11* | 0.0710 | 0.1057 | 0.1986 | 0.9011 | - | - | - |
| HSCL | 0.2811 | 0.0224* | 0.0321* | 0.0124* | 0.9434 | 0.9434 | - | - |
| Barrett | 0.9434 | 0.0259* | 2.3E-05* | 0.1182 | 0.9434 | 0.9434 | 0.9434 | - |
| Hoffer QST | 0.0090* | 3.7E-08* | 6.7E-08* | 0.0001* | 0.7076 | 0.8508 | 0.4493 | 0.0296* |

1. Comparisons of the standard deviation for the toric subgroup with adjusted *P*-values (heteroscedastic test and Holm correction)

| Formula | HTCL | Kane | EVO 2.0 | PEARL | HTAL | HSAL | HSCL | Barrett |
| --- | --- | --- | --- | --- | --- | --- | --- | --- |
| HTCL | - | - | - | - | - | - | - | - |
| Kane | 0.1204 | - | - | - | - | - | - | - |
| EVO 2.0 | 0.0941 | 0.9513 | - | - | - | - | - | - |
| PEARL | 0.1087 | 0.9513 | 0.9513 | - | - | - | - | - |
| HTAL | 0.E+00* | 0.9513 | 0.9513 | 0.9513 | - | - | - | - |
| HSAL | 1.6E-14* | 0.9513 | 0.9513 | 0.9513 | 0.9513 | - | - | - |
| HSCL | 1.1E-09* | 0.6345 | 0.4058 | 0.2838 | 0.0877 | 0.1732 | - | - |
| Barrett | 3.3E-06* | 0.0014* | 4.5E-09* | 0.0011* | 0.0407* | 0.0516 | 0.9513 | - |
| Hoffer QST | 3.6E-10* | 8.4E-07* | 4.9E-08* | 6.8E-05* | 4.0E-06* | 3.5E-06* | 0.0113* | 0.5196 |

The asterisks (*) represent significant differences between the formulas

compared.

AL, axial length; CMAL, Cooke-modified axial length; HSAL, Haigis formula, single-optimized and AL-applied; HSCL, Haigis formula, single-optimized and CMAL-applied;

HTAL, Haigis formula, triple-optimized and AL-applied; HTCL, Haigis formula, triple-optimized and CMAL-applied; EVO, Emmetropia Verifying Optical formula; Hoffer QST, Hoffer Q/Savini/Taroni formula; PEARL–DGS, Prediction Enhanced by Artificial Intelligence and output Linearization–Debellemanière, Gatinel, and Saad.


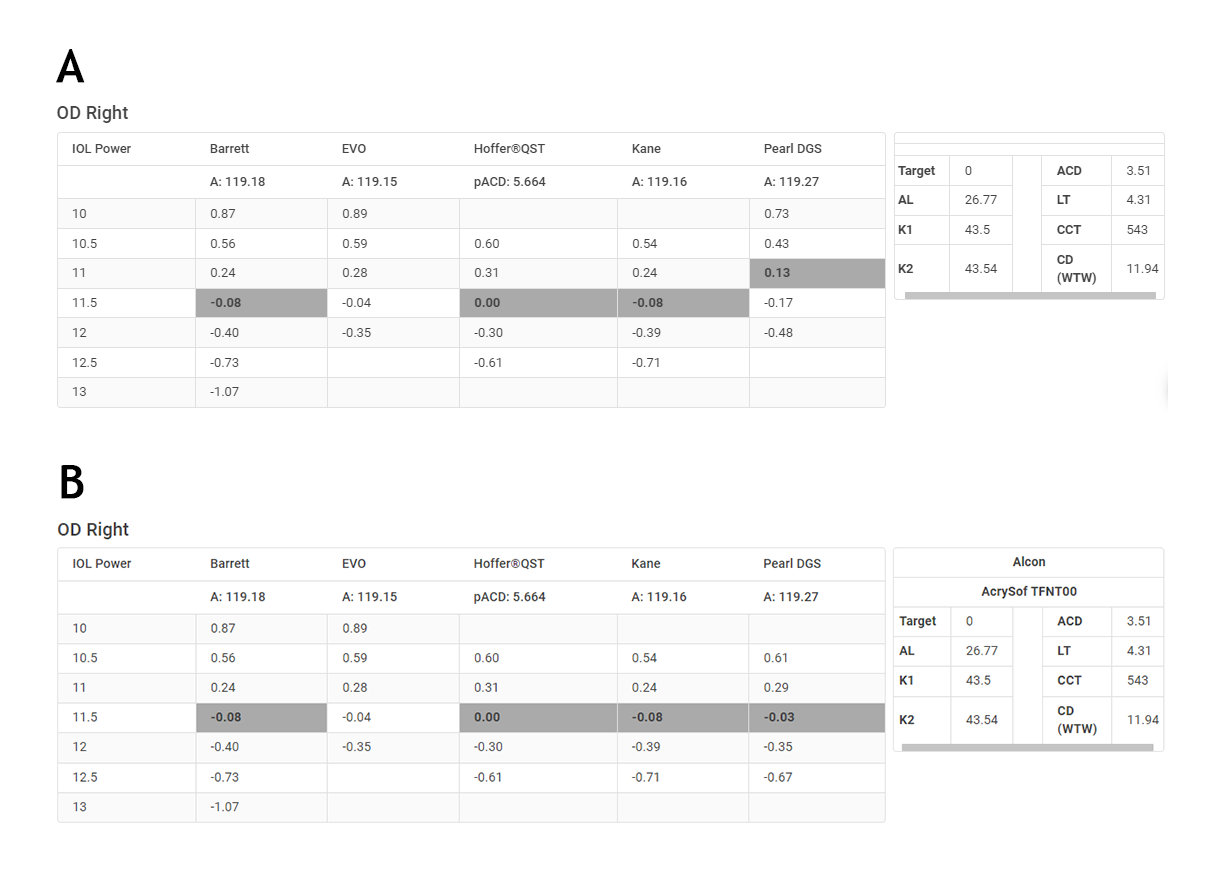


**Supplementary Fig. S1. The discrepancy in the predicted refraction with the same input data between the original PEARL-DGS online calculator (A) and the updated version (B), in comparison with the unchanged results of the other formulas.** (figure from the European Society of Cataract and Refractive Surgeons online calculator: https://iolcalculator.escrs.org/)

A, A constant; ACD, anterior chamber depth; AL, axial length; CCT, central corneal thickness; CD, corneal diameter; EVO, Emmetropia Verifying Optical formula; Hoffer QST, Hoffer Q/Savini/Taroni formula; IOL, intraocular lens; K1, keratometry of flat meridian; K2, keratometry of steep meridian; LT, lens thickness; pACD, personalized ACD; PEARL–DGS, Prediction Enhanced by Artificial Intelligence and output Linearization–Debellemanière, Gatinel, and Saad; WTW, white to white.
